# Minimum area thresholds for rattlesnakes and colubrid snakes on islands in the Gulf of California, Mexico

**DOI:** 10.1002/ece3.3658

**Published:** 2017-12-12

**Authors:** Jesse M. Meik, Robert Makowsky

**Affiliations:** ^1^ Department of Biological Sciences Tarleton State University Stephenville TX USA; ^2^ Department of Biostatistics University of Alabama at Birmingham Birmingham AL USA; ^3^ Almac Group Durham Durham NC USA

**Keywords:** Baja California, *Crotalus*, extinction risks, insular dwarfism, interspecific competition, island biogeography

## Abstract

We expand a framework for estimating minimum area thresholds to elaborate biogeographic patterns between two groups of snakes (rattlesnakes and colubrid snakes) on islands in the western Gulf of California, Mexico. The minimum area thresholds for supporting single species versus coexistence of two or more species relate to hypotheses of the relative importance of energetic efficiency and competitive interactions within groups, respectively. We used ordinal logistic regression probability functions to estimate minimum area thresholds after evaluating the influence of island area, isolation, and age on rattlesnake and colubrid occupancy patterns across 83 islands. Minimum area thresholds for islands supporting one species were nearly identical for rattlesnakes and colubrids (~1.7 km^2^), suggesting that selective tradeoffs for distinctive life history traits between rattlesnakes and colubrids did not result in any clear advantage of one life history strategy over the other on islands. However, the minimum area threshold for supporting two or more species of rattlesnakes (37.1 km^2^) was over five times greater than it was for supporting two or more species of colubrids (6.7 km^2^). The great differences between rattlesnakes and colubrids in minimum area required to support more than one species imply that for islands in the Gulf of California relative extinction risks are higher for coexistence of multiple species of rattlesnakes and that competition within and between species of rattlesnakes is likely much more intense than it is within and between species of colubrids.

## 1 INTRODUCTION

The Equilibrium Theory of Island Biogeography portrays patterns of species richness on islands as a dynamic equilibrium between colonization and extinction of biotic elements (MacArthur & Wilson, 1967). Species with mainland source populations are predicted to occur on islands where the time between immigration events is less than the persistence time, or time to extinction, both of which are influenced by island area and isolation (Lomolino, 1986, 2000). For example, increased island area reduces extinction risks by providing more available habitat and presumably increased environmental heterogeneity, while extinction risks may be ameliorated on less isolated islands owing to more frequent immigration from mainland source populations (i.e., rescue effects). Many factors can confound interpretations of insular distributions of taxonomic groups based on this framework, most important being the geographic idiosyncrasies of real island archipelagos (Lomolino, 2000). Nonetheless, logistic regressions of species occupancy patterns (i.e., presence or absence on islands) as a function of island area and isolation (“species incidence functions”) are often informative and have been used to estimate the minimum island area necessary for supporting populations, and to indirectly evaluate relative persistence abilities (i.e., converse of extinction risks) of species (Adler & Wilson, 1985; Diamond, 1975; Frick, Hayes, & Heady, 2008a; Peltonen & Hanski, 1991).

Continental or land‐bridge island systems with clear minimum area effects are often interpreted as being dominated by extinction dynamics, especially when the influence of isolation on species occurrences is shown to be negligible (Lomolino, 1986, 2000). In contrast, island systems that show effects of both island isolation and area on individual species occurrences are interpreted as being in equilibrium, because recurrent colonization counteracts the influence of extinction, particularly on larger and/or less isolated islands. Over a hundred islands and islets occur in the western Gulf of California, Mexico, and consist mostly of post‐Pleistocene land‐bridge fragments that are relatively close to the Baja California peninsula. Previous studies have demonstrated that extinction dynamics prevail in this island system for most taxonomic groups examined (e.g., bats, lizards, land birds) and that species assemblages could be described as supersaturated continental biotas that have been relaxing toward equilibrium states with island area since gradual eustatic sea level rise during the early Holocene (Case, 2002; Cody & Velarde, 2002; Frick, Hayes, & Heady, 2008b; Wilcox, 1978).

Rattlesnakes (Viperidae; *Crotalus*) are apex predators on most islands in the western Gulf of California and are often dwarfed in body size relative to mainland conspecifics (Boback, 2003; Case, 1978; Meik, Lawing, & Pires‐daSilva, 2010). Each of four species has been shown to have populations with small body size relative to mainland populations, and these populations may sometimes attain high densities, particularly on small islands (Klauber, 1949; Meik et al., 2012). Both diet alteration (diet switch from mammals on mainland to primarily lizards on islands) and strong intraspecific competition (diversion of limited energetic resources from growth to reproductive output) have been hypothesized to result in insular dwarfism among various insular populations of rattlesnake species in the Gulf of California (Boback, 2003; Case, 1978; Meik et al., 2010). In contrast, various colubrid snake species (Colubridae, sensu Pyron et al., 2011) also inhabit islands in the Gulf of California, but dwarfism has not been reported for any populations of these species.

In this study, we leveraged expectations from species–area relationships (i.e., increased area harbors increased species richness), and a natural extension of species incidence functions from binary to ordinal logistic regression, to compare biogeographic patterns between rattlesnakes and colubrid snakes on islands in the western Gulf of California. In particular, we define and empirically estimate minimum area thresholds for supporting single species as well as minimum area thresholds for supporting two or more species from each of these taxonomic groups, as these thresholds have the potential to inform different biogeographic patterns. By focusing on small island effects, we circumvent the problems of analyzing species–area curves, which are difficult to compare across groups when there exist great differences in species richness from available source pools (Scheiner, 2003; Tjorve, 2003); for our method, only two or more species are required from each mainland source pool to make valid comparisons across groups.

**Figure 1:**
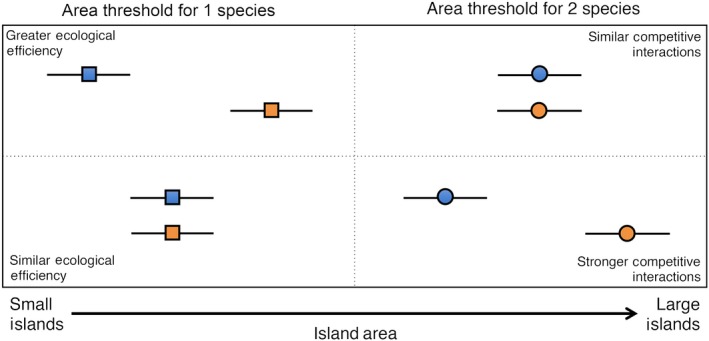
Diagram of hypothetical patterns that result from comparisons of minimum area thresholds for supporting single species (squares) and two species (circles) between two taxonomic groups (blue and orange). Differences in minimum area thresholds for supporting single species imply differences in ecological (energetic) efficiency and/or carrying capacity per unit area, whereas differences in minimum area thresholds for supporting coexisting species imply stronger competitive interactions within and between species of a group

Our conceptual framework provides different inferences for thresholds supporting one versus two or more species (Figure 1). The minimum area threshold for supporting a single species relates to ecological efficiency in this xeric island system. Because extinction risk is inversely related to population size, lower minimum area thresholds for a particular group of snakes would indicate higher carrying capacity (*K*), and thus greater absolute population size, for an island of particular surface area, and therefore implies greater energetic or ecological efficiency (MacArthur, Diamond, & Karr, 1972; MacArthur & Wilson, 1967). A lower minimum area threshold also means that more small islands are available for supporting populations long term and could therefore contribute to patterns of higher island incidence for a particular group. For example, a lower minimum area threshold for supporting a population of rattlesnakes could result from either greater colonization ability (i.e., overwater dispersal) or through greater long‐term persistence of island populations (i.e., lower extinction probabilities). As a group, rattlesnakes differ from most colubrid snakes from the region in that they produce large quantities of toxic venoms, and are heavy‐bodied ambush predators with low relative metabolic rates that eat large meals infrequently (Lillywhite & Smits, 1992; Secor & Diamond, 1998a,b). These traits may allow rattlesnakes to take advantage of large, temporarily available food supplies such as nestling sea birds or stranded or migrating passerine birds, as well as endure prolonged periods with limited food resources (McCue, 2007; Secor & Nagy, 1994). Rattlesnakes also exhibit traits that might result in higher capacity for overwater dispersal to islands, such as lower relative metabolic rates (confers fasting endurance), lower surface area‐to‐mass ratios (increases buoyancy for drifting snakes), and viviparity (allows pregnant females to retain embryos long enough to reach an island before parturition).

Differences between groups in the minimum area threshold for supporting two or more species relate to hypotheses of competitive interactions among phylogenetic lineages or otherwise ecologically similar species (Figure 1). High population densities are prevalent among reptiles inhabiting small islands generally (Hasegawa, 1994; Novosolov, Raia, & Meiri, 2013; Novosolov et al., 2016; Rodda & Dean‐Bradley, 2002) and likely lead to increased intraspecific competition and shifts in life history traits collectively termed the “island syndrome” (Adler & Levins, 1994; Novosolov & Meiri, 2013). High densities of individuals are presumed to occur because fewer competitor and predator species allow for resident species to each share a higher proportion of available resources (MacArthur et al., 1972; Pafilis, Meiri, Foufopoulos, & Valakos, 2009). In turn, high densities and strong intraspecific competition will simultaneously increase interspecific competition, leading to competitive exclusion of ecologically similar species. This phenomenon could manifest through higher minimum area thresholds for two or more species co‐occurring on an island, as more area would be required to support populations of strongly competing species over ecological timescales. For example, species of rattlesnakes might exhibit higher minimum area thresholds for supporting two or more species than do colubrid snakes, a pattern that would be consistent with the hypothesis that strong competitive interactions partly structure island occupancy of insular rattlesnakes.

## 2 MATERIALS AND METHODS

### 2.1 Study area and data collection

**Figure 2:**
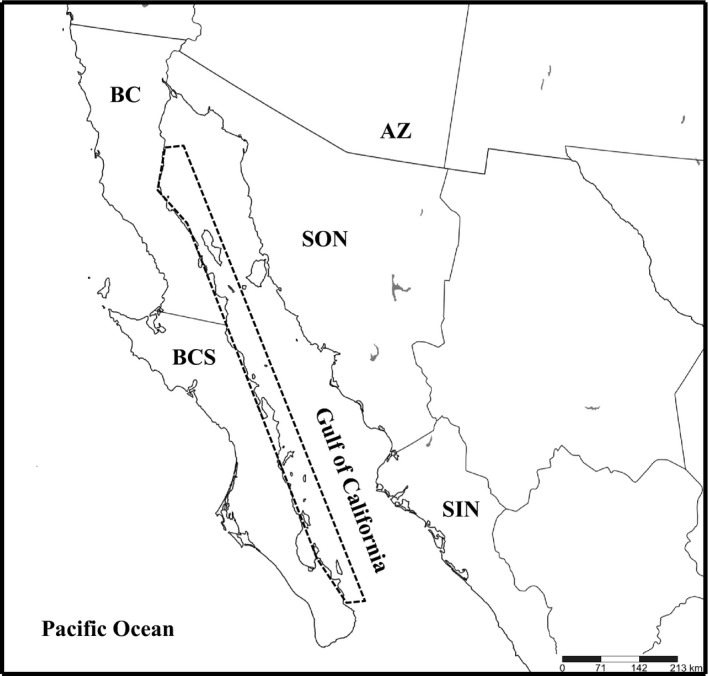
Map of western Mexico, including the Baja California Peninsula and Gulf of California. The shape outlined with dashed lines indicates the study region (islands of the western Gulf of California)

The Gulf of California is a narrow sea that extends approximately 1,200 km along a northwest to southeast axis, bordered on the east by the northwestern coast of mainland Mexico, and on the west by the Baja California peninsula (Figure 2). Numerous islands occur in the gulf, especially along the peninsular coastline, where they are situated in isolation or as subarchipelagos. The majority of these islands are believed to be land bridge in origin and shared a connection with the peninsula during the last glacial maximum between 16 and 24 ky; these islands have since been isolated in succession as sea levels rose during the early Holocene (Carreño & Helenes, 2002). The remaining islands likely are oceanic in origin or were sheared from the peninsula by tectonic activity within the past six my as the Baja California peninsula rifted toward the northwest, forming the Gulf of California (Londsdale, 1989). In general, islands in the Gulf of California are rugged and steep and characterized by Sonoran Desert vegetation (Cody, Moran, Rebman, & Thompson, 2002).

The snake fauna of the islands of the Gulf of California is well documented and has been subject of various biogeographic treatments (Case, 2002; Grismer, 2002; Murphy & Aguirre‐Léon, 2002). From these sources, we collated data on species occurrences (presence/absence) for all rattlesnakes and colubrid snakes, island area, and island isolation for 83 islands from the central and western Gulf of California (we collected additional information on island variables from Murphy, Sanchez‐Piñero, Polis, & Aalbu, 2002). Isolation was measured as distance from the peninsular mainland (the closest source pool) despite three islands inhabited by Western Diamondback rattlesnakes (*Crotalus atrox*)*,* which almost certainly originated from mainland Mexico. We considered all island populations as derivatives of their most closely related peninsular taxa, regardless of their status as unique species, as this conservative nomenclature does not affect our statistical analyses. Finally, we also collated data on island ages for as many islands in the gulf as possible; however, the dynamic geological history of the region has led to many controversial and conflicting dates for islands, and reliable estimates are few and restricted mostly to some land‐bridge fragments (Carreño & Helenes, 2002; Hurtado, Lee, & Mateos, 2013; Ledesma‐Vásquez & Carreño, 2010; Londsdale, 1989). Isolation, area, and age measurements for islands were log_10_‐transformed for all analyses. All data used for this study are provided as Table S1. All analyses were conducted in R version 3.2.1 (R Core Team, 2014).

### 2.2 Data analysis

Our method for estimating minimum area thresholds assumes that distribution patterns are driven by island area and are not confounded by the effects of island isolation or island age; therefore, we also evaluated the influence of these variables on rattlesnake and colubrid distribution patterns. Because we had matching (and complete) data for island area and isolation, we assessed the influence of these variables jointly using model selection approaches (see below). We evaluated the influence of island age on island occupancy as a separate regression analysis because data on island age were more limited in availability and reliability. Specifically, we regressed the residuals from the best rattlesnake and colubrid models (see paragraph below) on island age and looked for evidence of a relationship between the two variables.

We applied ordinal logistic regression with a cumulative logit link using the function *clm* in package “ordinal” version 2015.6‐28 (Christensen, 2015) to model the number of rattlesnake and colubrid species present on islands as a function of island area and isolation. This statistical approach takes advantage of ordered responses and assumes the same functional relationship between each state while also specifying the shift for each ordered transition. The ordered states were zero, one, and two or more species present with islands as the functional unit, implying that the shifts were from 0 to 1 species and 1–2 species present. We chose to bucket islands with two or more species because of the paucity of representation above two in rattlesnakes (and three in colubrids). Five a priori candidate models were developed with the following effects: area, isolation, area + isolation (additive effects), area*isolation (interaction, or compensatory effects), and random occurrence (Frick et al., 2008a; Lomolino, 2000). Models were estimated separately for rattlesnakes and colubrids. We selected the best‐fit models for each group using the Akaike information criterion (AIC) as recommended by Burnham and Anderson (2002). We ranked relative support for each model by comparing the best model (AIC_min_) and each competing model (AIC_*i*_) to find the ∆AIC. We then calculated the relative weights (AIC_W_), or likelihoods, for each model using the *akaike.weights* function in the “qpcR” package version 1.4‐0 (Spiess, 2015). To assess model fit, we calculated the pseudo‐*R* ^2^ of each model by comparing the log‐likelihood of each model to that of the random occurrence model (McFadden, 1974). For both rattlesnakes and colubrids, we evaluated model support separately for all five candidate models, and then for models that included only area, isolation, and random effects (see Results for reasoning). R code for ordinal logistic regression with cumulative probability functions is available as Data S1.

To empirically estimate minimum area thresholds, we extended the binary logistic regression analyses previously used to evaluate island occupancy of individual species in island systems (e.g., Diamond, 1975; Frick et al., 2008a; Lomolino, 2000) to an ordinal regression framework that bins species from each group to predict number of species as a function of island area. For each snake category, we interpreted two minimum area thresholds: (i) the point of intersection between the logistic probability functions where one species present exceeds the probability of zero species present, and (ii) the point of intersection between the logistic probability functions where two or more species present exceeds the probability of one species present (Figure S1). To find these intersections within R, we wrote a basic function that searched for the crossing points; the code for this function is available as Data S2. To assess variability in our minimum area threshold estimates, we conducted 10,000 bootstrap replicates where for each replicate islands were chosen with replacement and the crossing points were re‐estimated. We used the 0.025 and 0.975 quantiles from the 10,000 replicates to empirically estimate the 95% CI for both crossing points.

## 3 RESULTS

Results of model selection for the different combinations of main effects, additive effects, and interaction effects supported area as the dominant explanation of species incidence on islands in the Gulf of California for both rattlesnakes and colubrids (Table 1). When all five models were considered, neither the AIC relative likelihoods nor *R* ^2^ values found isolation to be associated with snake occurrence in any meaningful way. Specifically, the model with the lowest AIC value for both rattlesnakes and colubrids included only area, and the *R* ^2^ (and AIC) values barely changed when isolation was added to the area model. This effect was more obvious when only the area, isolation, and random occurrence models (main effects) were included. In this case, the relative likelihood of the area model was >99 times that of the isolation model (Table 1).

**Table 1:** Model selection results from ordinal logistic regression fitting number of species (0, 1, or 2+) as a function of island area (A) and isolation (I), including random occurrence effects as well as additive and interaction terms

Model	Groups	*K*	AIC	ΔAIC	AIC weights	AIC weights^a^	*R* ^2^
A	Rattlesnakes	3	62.27	0	0.64	>0.99	.57
	Colubrids	3	44.02	0	0.44	>0.99	.70
I	Rattlesnakes	3	127.12	64.85	<0.01	<0.01	.07
	Colubrids	3	121.51	76.49	<0.01	<0.01	.12
A + I	Rattlesnakes	4	64.17	1.90	0.25	NA	.57
	Colubrids	4	45.79	0.77	0.30	NA	.71
A + I + (A × I)	Rattlesnakes	5	65.87	3.60	0.11	NA	.57
	Colubrids	5	46.07	1.05	0.26	NA	.73
Random	Rattlesnakes	2	134.38	72.11	<0.01	<0.01	NA
	Colubrids	2	135.59	90.57	<0.01	<0.01	NA

a Akaike information criterion (AIC) weights recalculated using only area, isolation, and random effects.

Because effects of island area overwhelmed effects of island isolation in explaining snake occupancy patterns, we found very little evidence of island isolation as a confounding factor for estimating minimum area thresholds for either rattlesnakes or colubrids. While information on island age is scarce, we did look for an association between island age and number of species by calculating the residuals from the best rattlesnake and colubrid models and plotting them against island age for islands for which we had reliable data. The residuals were calculated as the difference in the observed number of species on an island and the state (0, 1, or 2 species) with the highest associated probability according to the area‐only model. While the sample size was small, we also observed minimal, if any, influence of island age on insular distribution patterns of snakes (Figure S2), indicating that island age also did not confound minimum area estimates.

**Figure 3:**
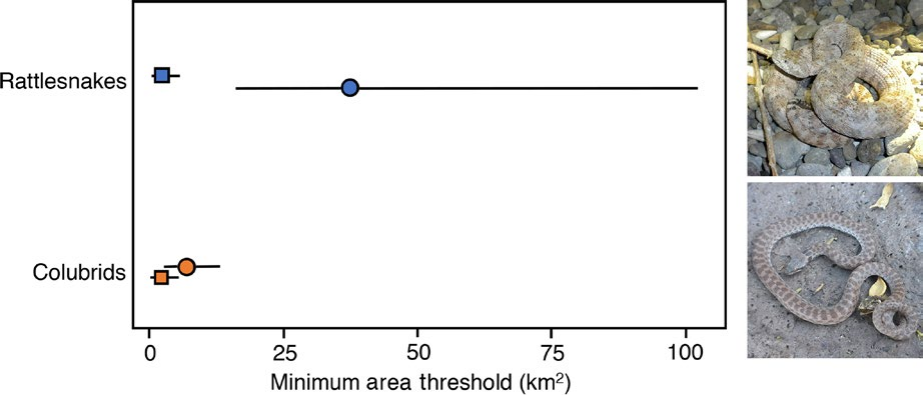
Empirical estimates of minimum area thresholds for supporting single species were nearly identical for rattlesnakes and colubrid snakes (1.8 vs. 1.7 km^2^, respectively), but area estimates for supporting two or more species of rattlesnakes were over five times greater than for colubrids (37.1 vs. 6.7 km^2^, respectively). Photographs depict an Angel de la Guarda speckled rattlesnake (*Crotalus angelensis*; top) and a California night snake (*Hypsiglena ochrorhyncha*; bottom), both from Angel de la Guarda Island

With respect to estimates of minimum area thresholds, the intersections of the logistic probability functions for zero to one species present were nearly identical between rattlesnakes and colubrids at 1.8 (95% CI: 0.9–3.9) and 1.7 (95% CI: 1.1–3.9) km^2^, respectively (Figure 3). However, the intersections of the logistic probability functions between one and two or more species present differed considerably between rattlesnakes and colubrids at 37.1 (95% CI: 16.3–102.4) and 6.7 (95% CI: 2.7–13.3) km^2^, respectively (Figure 3). Thus, to increase from one rattlesnake species to two or more species required over five times the area that was required to increase from one to two or more species of colubrids on islands in the western Gulf of California.

## 4 DISCUSSION

In this study, we used intersection points of probability functions generated from ordinal logistic regression models with a cumulative logit link to estimate the minimum area required for islands to support populations of one or more species of rattlesnakes and colubrid snakes. Although to our knowledge this method has not been used in this context, we believe it is justifiable, and our results should be reliable as long as effects of island isolation and age are not differentially influencing distribution patterns between rattlesnakes and colubrids. For example, greater overwater colonization abilities for one group of snakes could bias minimum area estimates downward, as propagules could become temporarily established on islands that are too small to support populations long term. Nonetheless, the strong influence of area and the negligible influence of island isolation and age on occupancy patterns provide evidence that these rescue effects have not notably influenced our estimates of minimum area thresholds.

Lower minimum area thresholds translate into potentially more islands available to support populations, and imply higher carrying capacity, and therefore greater ecological efficiency, for populations per unit area (MacArthur & Wilson, 1967). Assuming our estimates are reasonable for both groups of snakes, we found that minimum area thresholds for supporting one species were nearly identical for rattlesnakes and colubrids, indicating that neither group exhibits traits that could be interpreted as ecologically advantageous under the harsh and stochastic conditions of Gulf of California islands. Previously, we suggested some traits that might be advantageous for rattlesnakes in harsh insular environments, such as lower relative metabolic rates, production of toxic venoms, and viviparity. Rather, such traits should perhaps be viewed as tradeoffs that in some contexts might hinder long‐term persistence (Reed & Shine, 2002). While relative metabolic rates are lower for rattlesnakes (Lillywhite & Smits, 1992; Secor & Nagy, 1994), absolute metabolic rates are often higher owing to greater absolute mass, and while venom is useful for subduing large and potentially dangerous prey, the physiological costs of producing it could be prohibitive (McCue, 2006).

In contrast to the similarity of minimum area thresholds for single species, rattlesnakes required over five times the area as colubrids for islands to support an additional species (Figure 3). The most parsimonious explanation for this pattern is that rattlesnakes experience stronger competition, both within species and between species. Rattlesnake species tend to be more similar to each other in terms of ecology and life history traits than do species of colubrids, which would result in more intense competition for limiting resources. High densities of conspecifics could displace or resist invasion from ecologically similar species until islands reach a much greater threshold area, as we observe in rattlesnakes. Intense competition within and between rattlesnake species could also explain the pattern of insular dwarfism that is prevalent among rattlesnake populations on islands in the Gulf of California (Case, 1978; Meik et al., 2010). Although well documented among rattlesnake species, this phenomenon has not been reported for colubrids inhabiting islands in the region. Intraspecific competition in rattlesnakes may promote selection (or selection on plasticity) for diverting limited energetic resources from somatic growth to reproductive output, and lead to reduced adult body sizes in insular rattlesnake populations (Meik et al., 2010). Such a scenario is more likely to occur under relaxed predation pressures, which tend to happen on small and depauperate islands (Palkovacs, 2003).

## CONFLICT OF INTEREST

None declared.

## AUTHOR CONTRIBUTIONS

J.M.M. conceived the idea for the study and collated data for analyses, R.M. carried out statistical analyses, and both J.M.M. and R.M. wrote the manuscript.

## References

[ece33658-bib-0001] Adler, G. H. , & Levins, R. (1994). The island syndrome in rodent populations. The Quarterly Review of Biology, 69, 473–490. https://doi.org/10.1086/418744 7855232 10.1086/418744

[ece33658-bib-0002] Adler, G. H. , & Wilson, M. L. (1985). Small mammals on Massachusetts islands: The use of probability functions in clarifying biogeographic relationships. Oecologia, 66, 178–186. https://doi.org/10.1007/BF00379852 28311587 10.1007/BF00379852

[ece33658-bib-0003] Boback, S. M. (2003). Body size evolution in snakes: Evidence from island populations. Copeia, 2003, 81–94. https://doi.org/10.1643/0045-8511(2003)003[0081:BSEISE]2.0.CO;2

[ece33658-bib-0004] Burnham, K. P. , & Anderson, D. R. (2002). Model selection and multimodal inference: A practical information‐theoretic approach. New York, NY: Springer.

[ece33658-bib-0005] Carreño, A. L. , & Helenes, J. (2002). Geology and ages of the islands In Case T. J., Cody M. L., & Ezcurra E. (Eds.), A new island biogeography of the Sea of Cortés (pp. 14–40). New York, NY: Oxford University Press.

[ece33658-bib-0006] Case, T. J. (1978). A general explanation for insular body size trends in terrestrial vertebrates. Ecology, 59, 1–18. https://doi.org/10.2307/1936628

[ece33658-bib-0007] Case, T. J. (2002). Reptiles In Case T. J., Cody M. L., & Ezcurra E. (Eds.), A new island biogeography of the Sea of Cortés (pp. 221–270). New York, NY: Oxford University Press.

[ece33658-bib-0008] Christensen, R. H. B. (2015). Ordinal – Regression models for ordinal data. R package version 2015.6‐28. http://www.cran.r-project.org/package=ordinal/

[ece33658-bib-0009] Cody, M. L. , Moran, R. , Rebman, J. , & Thompson, H. (2002). Plants In Case T. J., Cody M. L., & Ezcurra E. (Eds.), A new island biogeography of the Sea of Cortés (pp. 63–111). New York, NY: Oxford University Press.

[ece33658-bib-0010] Cody, M. L. , & Velarde, E. (2002). Land birds In Case T. J., Cody M. L., & Ezcurra E. (Eds.), A new island biogeography of the Sea of Cortés (pp. 271–312). New York, NY: Oxford University Press.

[ece33658-bib-0011] Diamond, J. M. (1975). Assembly of species communities In Cody M. L. & Diamond J. M. (Eds.), Ecology and evolution of communities (pp. 342–444). Cambridge, MA: Harvard University Press.

[ece33658-bib-0012] Frick, W. F. , Hayes, J. P. , & Heady, P. A. III (2008a). Patterns of island occupancy in bats: Influences of area and isolation on insular incidence of volant mammals. Global Ecology and Biogeography, 17, 622–632. https://doi.org/10.1111/j.1466-8238.2008.00401.x

[ece33658-bib-0013] Frick, W. F. , Hayes, J. P. , & Heady, P. A. III (2008b). Island biogeography of bats in Baja Califorina, Mexico: Patterns of bat species richness in a near‐shore archipelago. Journal of Biogeography, 35, 353–364. https://doi.org/10.1111/j.1365-2699.2007.01798.x

[ece33658-bib-0014] Grismer, L. L. (2002). Amphibians and reptiles of Baja California, including its Pacific Islands and the Islands in the Sea of Cortés. Berkeley, CA: University of California Press https://doi.org/10.1525/california/9780520224179.001.0001

[ece33658-bib-0015] Hasegawa, M. (1994). Insular radiation in life history of the lizard *Eumeces okadae* in the Izu Islands, Japan. Copeia, 1994, 732–747. https://doi.org/10.2307/1447190

[ece33658-bib-0016] Hurtado, L. A. , Lee, E. J. , & Mateos, M. (2013). Contrasting phylogeography of sandy vs. rocky supralittoral isopods in the megadiverse and geologically dynamic Gulf of California and adjacent areas. PLoS One, 8, e67827 https://doi.org/10.1371/journal.pone.0067827 23844103 10.1371/journal.pone.0067827 PMC3699670

[ece33658-bib-0017] Klauber, L. K. (1949). Some new and revised subspecies of rattlesnakes. Transactions of the San Diego Society of Natural History, 11, 61–116. https://doi.org/10.5962/bhl.part.28857

[ece33658-bib-0018] Ledesma‐Vásquez, J. , & Carreño, A. L. (2010). Origin, age, and geological evolution of the Gulf of California In Brusca R. C. (Ed.), The Gulf of California: Biodiversity and conservation (pp. 7–23). Tucson, AZ: The University of Arizona Press.

[ece33658-bib-0019] Lillywhite, H. B. , & Smits, A. W. (1992). The cardiovascular adaptations of viperid snakes In Campbell J. A. & Brodie E. (Eds.), Biology of the pitvipers (pp. 143–153). Tyler, TX: Selva Press.

[ece33658-bib-0020] Lomolino, M. V. (1986). Mammalian community structure on islands: The importance of immigration, extinction, and interactive effects. Biological Journal of the Linnean Society, 28, 1–21. https://doi.org/10.1111/j.1095-8312.1986.tb01746.x

[ece33658-bib-0021] Lomolino, M. V. (2000). A species‐based theory of insular zoogeography. Global Ecology and Biogeography, 9, 39–58. https://doi.org/10.1046/j.1365-2699.2000.00188.x

[ece33658-bib-0022] Londsdale, P. (1989). Geology and tectonic history of the Gulf of California In Winter E. L., Hussong D. M., & Decker R. W. (Eds.), The Geology of North America Series: The Eastern Pacific Ocean and Hawaii (pp. 499–521). Boulder, CO: Geological Society of America.

[ece33658-bib-0023] MacArthur, R. H. , Diamond, J. M. , & Karr, J. R. (1972). Density compensation in island faunas. Ecology, 53, 330–342. https://doi.org/10.2307/1934090

[ece33658-bib-0024] MacArthur, R. H. , & Wilson, E. O. (1967). The theory of island biogeography. Princeton, NJ: Princeton University Press.

[ece33658-bib-0025] McCue, M. D. (2006). Cost of producing venom in three North American pitviper species. Copeia, 2006, 818–825. https://doi.org/10.1643/0045-8511(2006)6[818:COPVIT]2.0.CO;2

[ece33658-bib-0026] McCue, M. D. (2007). Western diamondback rattlesnakes demonstrate physiological and biochemical strategies for tolerating prolonged starvation. Physiological and Biochemical Zoology, 80, 25–34. https://doi.org/10.1086/509057 17160877 10.1086/509057

[ece33658-bib-0027] McFadden, D. (1974). Conditional logit analysis of qualitative choice behavior In Zarembka P. (Ed.), Frontiers in econometrics (pp. 105–142). Cambridge, MA: Academic Press.

[ece33658-bib-0028] Meik, J. M. , Lawing, A. M. , & Pires‐daSilva, A. (2010). Body size evolution in insular speckled rattlesnakes. PLoS One, 5, e9524 https://doi.org/10.1371/journal.pone.0009524 20209105 10.1371/journal.pone.0009524 PMC2832004

[ece33658-bib-0029] Meik, J. M. , Schaack, S. , Ingrasci, M. J. , Setser, K. , Mociño‐Deloya, E. , & Flores‐Villela, O. (2012). Notes on activity, body size variation, and diet in insular speckled rattlesnakes from the western Sea of Cortés, Mexico. Herpetological Review, 43, 556–560. https://www.researchgate.net/publication/280026904

[ece33658-bib-0030] Murphy, R. W. , & Aguirre‐Léon, G. (2002). Distributional checklist of nonavian reptiles and amphibians on the islands in the Sea of Cortés In Case T. J., Cody M. L., & Ezcurra E. (Eds.), A new island biogeography of the Sea of Cortés (pp. 580–594). New York, NY: Oxford University Press.

[ece33658-bib-0031] Murphy, R. W. , Sanchez‐Piñero, F. , Polis, G. A. , & Aalbu, R. L. (2002). New measurements of area and distance for islands in the Sea of Cortés In Case T. J., Cody M. L., & Ezcurra E. (Eds.), A new island biogeography of the Sea of Cortés (pp. 447–464). New York, NY: Oxford University Press.

[ece33658-bib-0032] Novosolov, M. , & Meiri, S. (2013). The effect of island type on lizard reproductive traits. Journal of Biogeography, 40, 2385–2395. https://doi.org/10.1111/jbi.12179

[ece33658-bib-0033] Novosolov, M. , Raia, P. , & Meiri, S. (2013). The island syndrome in lizards. Global Ecology and Biogeography, 22, 184–191. https://doi.org/10.1111/j.1466-8238.2012.00791.x

[ece33658-bib-0034] Novosolov, M. , Rodda, G. H. , Feldman, A. , Kadison, A. E. , Dor, R. , & Meiri, S. (2016). Power in numbers. Drivers of high population density in insular lizards. Global Ecology and Biogeography, 25, 87–95. https://doi.org/10.1111/geb.12390

[ece33658-bib-0035] Pafilis, P. , Meiri, S. , Foufopoulos, J. , & Valakos, E. (2009). Intraspecific competition and high food availability are associated with insular gigantism in a lizard. Naturwissenschaften, 96, 1107–1113. https://doi.org/10.1007/s00114-009-0564-3 19488731 10.1007/s00114-009-0564-3

[ece33658-bib-0036] Palkovacs, E. P. (2003). Explaining adaptive shifts in body size on islands: A life history approach. Oikos, 103, 37–44. https://doi.org/10.1034/j.1600-0706.2003.12502.x

[ece33658-bib-0037] Peltonen, A. , & Hanski, I. (1991). Patterns of island occupancy explained by colonization and extinction rates in shrews. Ecology, 72, 1698–1708. https://doi.org/10.2307/1940969

[ece33658-bib-0038] Pyron, R. A. , Burbrink, F. T. , Colli, G. R. , Nieto Montes de Oca, A. , Vitt, L. J. , Kuczynski, C. A. , & Wiens, J. J. (2011). The phylogeny of advanced snakes (Colubroidea), with discovery of a new subfamily and comparison of support methods for likelihood trees. Molecular Phylogenetics and Evolution, 58, 329–342. https://doi.org/10.1016/j.ympev.2010.11.006 21074626 10.1016/j.ympev.2010.11.006

[ece33658-bib-0039] Reed, R. N. , & Shine, R. (2002). Lying in wait for extinction: Ecological correlates of conservation status among Australian elapid snakes. Conservation Biology, 16, 451–461. https://doi.org/10.1046/j.1523-1739.2002.02283.x

[ece33658-bib-0040] Rodda, G. H. , & Dean‐Bradley, K. (2002). Excess density compensation of island herpetofaunal assemblages. Journal of Biogeography, 29, 623–632. https://doi.org/10.1046/j.1365-2699.2002.00711.x

[ece33658-bib-0041] Scheiner, S. M. (2003). Six types of species‐area curves. Global Ecology and Biogeography, 12, 441–447. https://doi.org/10.1046/j.1466-822X.2003.00061.x

[ece33658-bib-0042] Secor, S. M. , & Diamond, J. M. (1998a). A vertebrate model of extreme physiological regulation. Nature, 395, 659–662. https://doi.org/10.1038/27131 9790187 10.1038/27131

[ece33658-bib-0043] Secor, S. M. , & Diamond, J. M. (1998b). Evolution of regulatory responses to feeding in snakes. Physiological and Biochemical Zoology, 73, 123–141. https://doi.org/10.1086/316734 10.1086/316734 10801391

[ece33658-bib-0044] Secor, S. M. , & Nagy, K. A. (1994). Bioenergetic correlates of foraging mode for the snakes *Crotalus cerastes* and *Masticophis flagellum* . Ecology, 75, 1600–1614. https://doi.org/10.2307/1939621

[ece33658-bib-0045] Spiess, A. N. (2015). qpcR: Modelling and analysis of real‐time PCR data. R package version 1.4‐0. https://cran.r-project.org/web/packages/qpcR/

[ece33658-bib-0046] Tjorve, E. (2003). Shapes and functions of species‐area curves: A review of possible models. Journal of Biogeography, 30, 827–835. https://doi.org/10.1046/j.1365-2699.2003.00877.x

[ece33658-bib-0047] Wilcox, B. A. (1978). Supersaturated island faunas: A species‐age relationship for lizards on post‐Pleistocene land‐bridge islands. Science, 199, 996–998. https://doi.org/10.1126/science.199.4332.996 17752374 10.1126/science.199.4332.996

