# Supplementary material for: Minimum area thresholds for rattlesnakes and colubrid snakes on islands in the Gulf of California, Mexico
Source: Ecol Evol. 2017 Dec 12;8(2):928–34. doi: 10.1002/ece3.3658 (PMC5773319; doi:10.1002/ece3.3658)
Supplement: Supplementary file 3 [file ECE3-8-928-s003.docx]

**Online Supplemental File S3**. Supplemental figures.

Fig. S1. Ordinal logistic probability functions modeling number of snake species present on islands in the western Gulf of California; (A) number of rattlesnake species as a function of island area (log_10_ km^2^), (B) number of rattlesnake species as a function of island isolation (log_10_ km), (C) number of colubrid species as a function of island area (log_10_ km^2^), and (D) number of colubrid species as a function of island isolation (log_10_ km).

Fig. S2. Association of residuals from the ordinal logistic regression (area-only models) for rattlesnakes and colubrids and island age for islands with available data.
